# Esophageal Gastrointestinal Stromal Tumor With *MKRN1-BRAF* Fusion: A Rare Mediastinal Case

**DOI:** 10.1016/j.atssr.2025.11.021

**Published:** 2025-12-17

**Authors:** Bradley Kruithoff, Lauren Beard, David Liebner, Swati Satturwar, Matthew Lamb

**Affiliations:** 1 Department of Surgery, OhioHealth Doctors Hospital, Columbus, Ohio; 2 Department of Surgery, OhioHealth Grant Medical Center, Columbus, Ohio; 3 Ohio University Heritage College of Osteopathic Medicine, Athens, Ohio; 4 Department of Medical Oncology and Pathology, Wexner Medical Center and James Cancer Center, The Ohio State University, Columbus, Ohio; 5 Department of Cardiothoracic Surgery, OhioHealth Grant Medical Center, Columbus, Ohio

## Abstract

Gastrointestinal stromal tumors (GISTs) are rare mesenchymal neoplasms typically involving the upper gastrointestinal tract. Most GISTs are driven by activating mutations in the *KIT* or *PDGFRA* genes, which are primary molecular markers for diagnosis and treatment. In addition, CD117 and DOG1 (discovered on GIST 1) are important immunohistochemical diagnostic markers of GISTs. Esophageal GISTs are exceptionally rare, and those driven by *BRAF* gene fusions are even more uncommon. This report describes a 69-year-old woman who underwent surgical resection of an esophageal GIST harboring an *MKRN1-BRAF* gene fusion, offering insight into future treatment and surveillance strategies.

Gastrointestinal stromal tumors (GISTs), although rare, are the most common type of gastrointestinal mesenchymal tumors. GISTS are typically located in the stomach and small intestine, with only about 1% to 10% occurring in the esophagus, colon, or rectum.^1^^,^^2^ Although most GISTs are driven by *KIT* or *PDGFRA* gene mutations, our case of an *MKRN1-BRAF* gene fusion represents an extremely rare clinical finding that has been scarcely documented in the literature.

A 69-year-old woman presented to the thoracic surgery office with unintentional weight loss and anemia. Computed tomography imaging revealed a 2.6 × 2.7-cm paraesophageal mass with areas of decreased attenuation and necrosis, and subsequent positron emission tomography demonstrated an avid mass without associated mediastinal lymphadenopathy (Figures 1, 2). Bronchoscopy demonstrated normal bronchial anatomy, and subsequent esophagogastroduodenoscopy with endoscopic ultrasound and fine-needle aspiration demonstrated extrinsic compression of the distal esophagus in the area adjacent to the lesion. Preliminary cytologic examination of the fine-needle aspiration specimen was consistent with a spindle cell neoplasm. Upfront esophagectomy was thought to be unnecessary, given the uncertainty of diagnosis. Instead, the patient was consented for excisional biopsy to determine the exact pathologic process. If final pathologic examination was concerning, there were plans for multidisciplinary collaboration to discuss esophagectomy with potential neoadjuvant therapies. The patient subsequently underwent robot-assisted esophageal mass resection approached from the left side of the chest. This was completed through a thoracoscopic 4-port technique in a vertical hockey-stick fashion similar to the chest portion of an Ivor-Lewis esophagectomy. In addition, an assistant port was placed for specimen extraction. The mass was adherent to both the esophagus and the left lower lobe and was carefully mobilized and resected from the surrounding structures without violation of the capsule. The operation was tolerated well, and the patient was discharged home on postoperative day 2.

**Figure 1:**
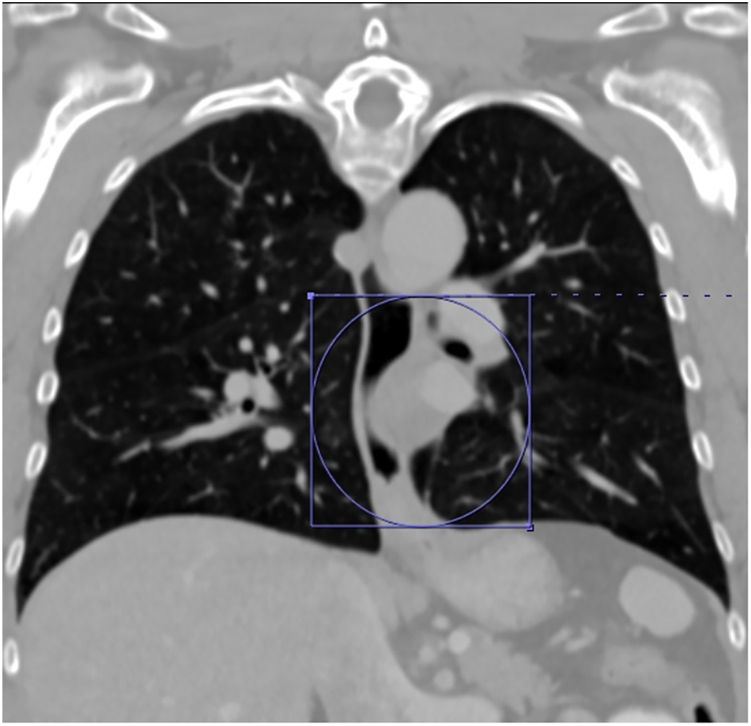
Computed tomography of the chest with intravenous administration of contrast material demonstrating a smoothly marginated 2.6 × 2.7-cm paraesophageal mass to the left of the midesophagus with extrinsic compression of the esophagus (mass shown within circle).

**Figure 2:**
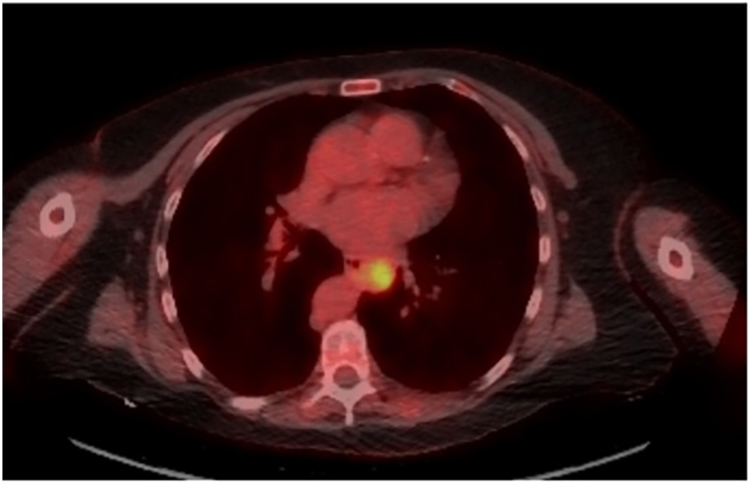
Metabolic activity associated with the paraesophageal mass without other metabolically active disease.

Official surgical pathologic examination demonstrated a well-circumscribed low-grade spindle cell neoplasm with myxoid stroma (Figure 3), with all sampled lymph nodes negative for malignancy. The mitotic count was 0 to 1 per 50 high-power fields. Tumor necrosis was not present. On immunohistochemical analysis, the tumor cells were positive for DOG1, CD34, SMA, and desmin (focal) and negative for CD117, STAT6, S100, SOX10, EMA, and ALK. Given the unusual location, unusual morphologic appearance with myxoid stroma, and immunoprofile with CD117 negativity, this case was initially signed out as low-grade spindle cell neoplasm with myxoid stroma, not further classifiable. Next-generation sequencing assay detected a somatic *MKRN1-BRAF* chromosomal rearrangement, confirming the diagnosis of GIST with *BRAF* fusion. Overall, given the small tumor size (<5 cm) and low mitotic count (<0-1 per 50 high-power fields), the tumor was considered at low risk of recurrence per the Miettinen classification,^3^ which is supported by the College of American Pathologists risk stratification criteria for GISTs.

**Figure 3:**
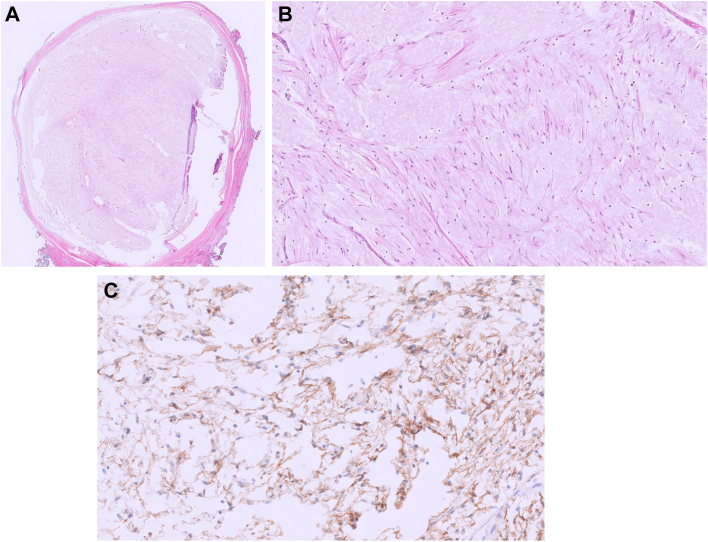
Esophageal gastrointestinal stromal tumor with *BRAF* fusion. (A) Low-magnification view of well-circumscribed nodule, showing (B) hypocellular cytology bland spindle cell proliferation with myxoid stroma and (C) positivity for DOG1. The tumor cells were negative for CD117 (not shown).

## Comment

Esophageal GISTs are extremely rare and account for <1% of GISTs. Whereas most GISTs express mutations in the *KIT* or *PDGFRA* genes, a small subset is characterized by other oncogenic drivers, such as *BRAF* mutations and fusions.

The pathologic finding of the *MKRN1-BRAF* translocation represents an exceedingly rare genomic finding. BRAF is a serine-threonine kinase in the RAF family of kinases and is a central component of the RAS/MAPK pathway involved in cell differentiation, proliferation, migration, and apoptosis. The fusion of *BRAF* with different 5′ partners, in this case *MKRN1*, replaces the *BRAF* autoregulatory domain with a dimerization domain from the partner gene, which results in the production of constitutively active *BRAF* homodimers that are capable of driving tumor progression.

*BRAF*-mutated GISTs are morphologically similar to and immunohistochemically different from *KIT*-mutated GISTs. On the basis of limited data, CD117 can be negative or weakly positive in *BRAF*-mutated GISTS. For *KIT*-mutated cases, all would be positive for CD117. Negativity for CD117 can pose diagnostic challenges. Given this, CD117 negativity and abundant myxoid stroma should suggest unusual molecular subtypes of GISTs. In working up spindle cell neoplasms, adding DOG1 to CD117 and other immunohistochemical markers, such as CD34, SMA, desmin, S100, SOX10, SS18, or STAT6, can improve diagnostic accuracy^4^

From a clinical perspective, the patient’s *MKRN1-BRAF* fusion–positive GIST would fall under the wild-type category, known to be resistant to standard tyrosine kinase inhibitors such as imatinib. These GISTs do not respond to standard tyrosine kinase inhibitor therapy because the *BRAF* oncogenic signal originates downstream of *KIT*. Therefore, even if imatinib blocks KIT completely, the BRAF-MEK-ERK pathway remains fully active because of the constitutively active *BRAF* mutation. There are several Food and Drug Administration–approved BRAF and MEK1/2 inhibitors on the market for treatment of *BRAF* or *MEK* mutation-positive cancers, and they are often used in combination to increase treatment efficacy.^5^

In respect to tumor immunohistochemistry in the context of prognosis and surveillance, because *BRAF*-mutant GISTs are rare, current literature is insufficient to assess impact on prognosis. Given the rarity of *BRAF* fusion GISTs, primary drivers of recurrence and stratification should be based on established risk stratification, such as size, mitotic count, and location. Unusual locations for GISTs, such as esophagus, mesentery, or peritoneum, should be classified according to the criteria for jejunal and ileal GISTs. As a result, this patient will undergo surveillance per institutional standard of care with restaging computed tomography of the chest, abdomen, and pelvis every 6 months for 24 months, followed by annual imaging through year 5 because of the low-risk tumor profile.

This report illustrates unusual morphology and the immunohistochemical variability seen in *BRAF*-mutated GISTs, emphasizing that all surgical pathology specimens, especially from unusual locations, should undergo comprehensive molecular profiling for accurate diagnosis and potential use of targeted therapy. Identifying actionable targets such as *MKRN1-BRAF* fusions may open opportunities for targeted therapy, improve postoperative surveillance strategies, and contribute to a growing understanding of GIST heterogeneity. In conclusion, we present the case of a patient with a paraesophageal GIST that was revealed to harbor an incredibly rare *MKRN1-BRAF* translocation mutation. To date, only a handful of GISTs with *BRAF* fusions and even fewer with esophageal involvement have been identified and documented in the literature. This report serves to add to the growing body of literature regarding fusion proteins and cellular signaling pathways and to help guide future clinicians with treatment and surveillance strategies when faced with unique tumor pathology.
